# Supplementary material for: CEP peptide and cytokinin pathways converge on CEPD glutaredoxins to inhibit root growth
Source: Nat Commun. 2023 Mar 27;14:1683. doi: 10.1038/s41467-023-37282-6 (PMC10042822; doi:10.1038/s41467-023-37282-6)
Supplement: Supplementary file 3 — Description of Additional Supplementary Files [file 41467_2023_37282_MOESM3_ESM.pdf]

Description of Additional Supplementary Files:

Supplementary Data 1: Cytokinin metabolite levels in roots and shoots of wild type and cepr1 plants
